# Bio-Oil Production from Date Palm Surface Fibers: Thermo-Kinetic and Pyrolysis GC/MS Analysis

**DOI:** 10.1021/acsomega.5c11064

**Published:** 2026-01-07

**Authors:** Abrar Inayat, Mohsin Raza, Labeeb Ali, Mohammednoor Altarawneh, Chaouki Ghenai, Farrukh Jamil, Abdallah Shanableh, Peer Mohamed Abdul, Faisal Mehmood Shah

**Affiliations:** † Research Institute of Science and Engineering, Biomass and Bioenergy RG, 59105 University of Sharjah, Sharjah 27272, United Arab Emirates; ‡ Department of Mechanical and Nuclear Engineering, University of Sharjah, Sharjah 27272, United Arab Emirates; § Petroleum Engineering Technology Department, Abu Dhabi Polytechnic University, Abu Dhabi 111499, United Arab Emirates; ∥ Chemical and Petroleum Engineering Department, College of Engineering, United Arab Emirates University, Al Ain 15551, United Arab Emirates; ⊥ Department of Sustainable and Renewable Energy Engineering, College of Engineering, University of Sharjah, Sharjah 27272, United Arab Emirates; # Department of Chemical Engineering, Faculty of Engineering and Technology, Muscat University, Muscat P.C.130, Oman; ¶ Research Institute of Sciences and Engineering (RISE), University of Sharjah, Sharjah 27272, United Arab Emirates; ∇ Department of Chemical and Process Engineering, Faculty of Engineering and Built Environment, Universiti Kebangsaan Malaysia, Bangi, Selangor 43600, Malaysia; ○ College of Engineering and Physical Sciences, 1722 Aston University, Birmingham B4 7ET, U.K.

## Abstract

Date palm surface fibers (DPSFs) are abundantly available as municipal and agricultural biomass wastes from date palm trees, especially in the Middle Eastern and North African countries, especially United Arab Emirates. DPSFs are lignocellulosic in nature and therefore have immense potential to be used for bioenergy purposes. This study presents the conversion-dependent pyrolysis behavior, kinetic analysis, and bio-oil qualitative investigation. DPSFs were analyzed using thermogravimetric analysis at nonisothermal heating rates of 10–40 °C/min at a temperature range of 20–750 °C. Activation energy (*E*_a_) was calculated using model-free kinetics approach using Ozawa–Flynn–Wall (OFW), Kissinger–Akahira–Sunose (KAS), and Starink (STK) methods. *E*_a_ analysis helps understand the link up of degradation behavior as a function of the conversion and fragmentation of cellulose, hemicellulose, and lignin. The pyrolysis of DPSFs was performed in a horizontal quartz tube flow reactor at a heating rate of 40 °C/min and within a temperature range of 20–400 °C. The condensed bio-oil was tested for qualitative analysis using a gas chromatography and mass spectroscopy (GC/MS) technique. *E*_a_ values for the active pyrolysis region within a conversion range of 0.2–0.8 were 154.52, 152.40, and 152.37 kJ/mol for the OFW, KAS, and STK models, respectively. Using GC/MS, the qualitative assessment of bio-oil, based on normalized peak area percentages, showed that it consisted mainly of aliphatics (42.28%), aromatics (38.68%), and furans/other oxygenates (13.47%). 10.54% benzene and 10.94% toluene were the main contributors of aromatics, and 6.735 furfural was a dominant furanic compound. The result of this study provides an information on the composition of DPSF pyrolyzed bio-oil and suggests that the aromatic rich nature will lead to targeted recovery of BTX/phenolics compounds as well as for bioenergy applications.

## Graphical abstract

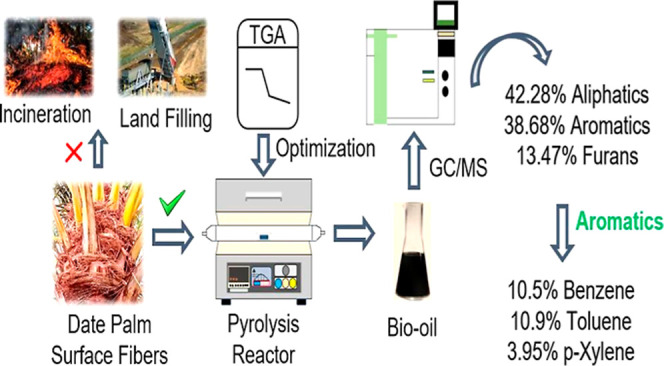

## 1 Introduction

Since the start of the Industrial Revolution, the energy demands have primarily been met using fossil fuels, such as petroleum, coal, and natural gas. To date, despite being recognized as less environmentally friendly, these resources dominate the energy market.^1^ Further estimation of their excessive exhaustion due to the rising pace of consumption is urging the global scientific community and industrial partners to look for further penetration of renewable sources into the energy mix.^2^ Renewable resources of energy production are also important to address the rising threats due to global warming, as indicated by the Intergovernmental Panel on Climate Change (IPCC).^3^ The IPCC special report on climate change indicates that a 1.5 °C rise in temperature, which is above preindustrial levels, should be seriously addressed.^4^

Lignocellulosic biomass waste is one of the most abundant renewable sources that can be converted into gaseous, liquid, and solid fuels.^5^ A recent study by Oak Ridge National Laboratory, USA, reports a total of 2.83 billion dry metric tons of biomass in 62 sample countries.^6^ Lignocellulose biomass consists of 35–50% cellulose, 20–35% hemicellulose, 10–20% lignin, and remaining extractives.^7^ Utilization of lignocellulose biomass waste promotes the concept of the circular economy, where waste is converted into renewable fuel.^8^ Pyrolysis, which is a thermochemical conversion technology, appears to be the most efficient and reliable process of converting lignocellulosic biomass into different fuels.^9^ Bio-oil production using pyrolysis offers advantages of improved energy efficiency, reduced CO_2_ emissions, low-cost, and a sustainable source of production.^10^

Bharath et al.^11^ conducted the fast pyrolysis of date seeds using a bench-scale fluidized bed reactor under optimized reaction conditions. The date seed pyrolysis provides a yield of 68 wt % bio-oil at a temperature of 500 °C, residence time of 30 min, biomass loading of 200 g, and 10 mL/min of fluidizing gas flow rate. The qualitative analysis of the biomass showed that date seed bio-oil had potential of high value fuel due to the presence of 2-furanmethanol, *d*-allose, and catechol. Joardder et al.^12^ performed the pyrolysis of date seeds in a fixed bed reactor. The bio-oil yield was 50 wt % at a reactor bed temperature of 500 °C and a total running time of 120 min. It was found that the bio-oil had a high calorific value (28.63 MJ/kg). Asadullah et al.^13^ performed the pyrolysis of palm kernel shell in a fluidized bed reactor. The bio-oil yield obtained was 57% at a temperature of 550C and 10 g/min biomass feed rate. It was found that the bio-oil from palm kernel shell waste provided a more than 40% area ratio of phenols in the GC analysis.

The United Arab Emirates is home to 42 million date palm trees (*Phoenix dactylifera L.*).^14^ Each tree is assessed to produce 20 kg of lignocellulose biomass waste annually in the form of date fruit seeds, leaves, fronds, and surface fibers.^15^ Suitable disposal of these million tons of lignocellulosic biomass waste requires an expensive waste management system. If not handled correctly by the municipalities, the incineration or burning in the field causes environmental dangers of greenhouse gases such as CO_2_ and nitric oxides (NO_*x*_).^16^ Utilizing lignocellulosic wastes also aligns with the holistic philosophy of permaculture, where nothing is considered a waste, instead a feedstock for a new product.^17^

Date palm surface fibers (DPSFs) are lignocellulosic waste that grows on top of the date palm tree. DPSFs are fibrous, lightweight, and rich in lignin and cellulose, which makes them highly suitable as a feedstock for pyrolysis to produce bio-oil and biochar.^18^ DPSFs have mainly been reported in the literature as a renewable source of producing thermal insulation material in combination with different polymers, such as PVA^19^ and polystyrene.^20^ Although DPSFs are easy to collect and eradicate the challenges related to biomass segregation, they remain largely underutilized as potential feedstocks for pyrolysis. Previously, Raza et al.^21^ performed the kinetic and thermodynamic analyses of DPFs using a model-fitting approach to highlight it as a feedstock for bioenergy. However, this study did not extend to experimental pyrolysis, leaving the characterization and evaluation of the resulting bio-oil products unknown.

Utilizing local lignocellulosic biomass waste also aligns strongly with the UAE’s national agenda to treat 80% of municipal solid waste by 2031 and Abu Dhabi’s emirate plans to divert 80% of solid biomass waste from landfilling by 2023.^22^ Utilizing biomass waste also aligns with the UAE’s national policy 2024 on biofuels. This policy provides a framework for producing sustainable, renewable fuels from biomass wastes.^23^ Different date palm residues, such as seeds, rachis, leaflets, stems, and pomace, have been widely used for bio-oil production via pyrolysis.^24–26^ However, feedstock-resolved compositional data for DPSFs remain limited. The linkage between conversion-dependent kinetics and the qualitative analysis of bio-oil products is also not available in the literature. In comparison to other date wastes, DPSFs are also easier to segregate and collect, as they appear at the top of the trunk, unlike other palm waste, which appear heterogeneously.^27^

To the best of the authors’ knowledge, this study presents the first experimental pyrolysis of DPSFs as a feedstock. The bio-oil was subsequently characterized through GC/MS analysis for a qualitative analysis of the product distribution. To find the optimum pyrolysis temperature range of the active pyrolysis region, TGA and DTG were performed at different heating rates (10, 20, 30, and 40 °C/min). Model-free isoconversional kinetics using Ozawa–Flynn–Wall (OFW), Kissinger–Akahira–Sunose (KAS), and Starink (STK) were performed to quantify the conversion-dependent degradation behavior. These methods are recommended by the ICTAC Kinetics Committee for their reliability in kinetic studies.^28^ The results of this study show that aromatic-rich bio-oil can be used for bioenergy and to selectively recover BTX and phenolic fractions.

## 2 Materials and Methods

### 2.1 Raw Materials

DPSFs were received from the palm trees at the University of Sharjah, Sharjah, UAE. The DPSFs were cleaned with compressed air to remove any dust and contaminants. DPSFs were then reduced to a mesh size of 120 μm with laboratory grinders (ultracentrifugal mill Z 200). Before any analysis, DPSFs were heated to 105 °C for 24 h to reduce moisture. Figure 1 shows the raw DPSFs and their SEM image. Raza et al.^19^ used DPSFs used for developing thermal insulation materials. FTIR analysis of the DPSFs confirms the lignocellulosic nature of these fibers.

**Figure 1:**
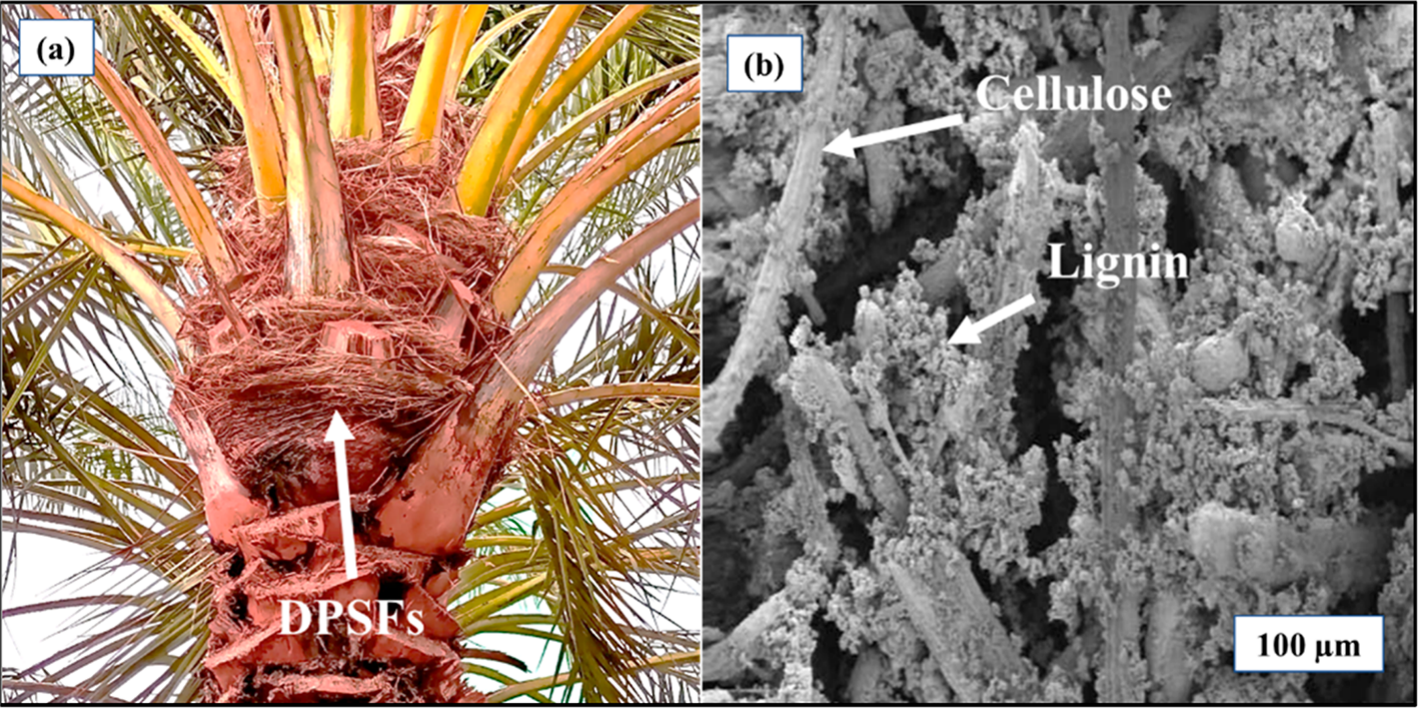
(a) Raw DPSFs and (b) SEM image of DPSFs.

### 2.2 Physiochemical Characterizations

Proximate analysis was performed using a standard procedure of ASTM D3172-07a, and the CHNS analyzer was used to perform the ultimate analysis. Moisture contents of the DPSFs were measured without oven drying to check the moisture level without any processing. Calorific value was measured using a Parr 6400 Calorimeter. In chemical composition analysis, the weight percentages of cellulose, hemicellulose, and lignin were calculated using standard ASTM (D1104-56, D1103-0, and D1104-56) and TAPPI (T 204 cm-97) protocols. Details of the procedures are available in our previous publications.^29^

### 2.3 Thermogravimetric Analysis­(TGA)

The thermal degradation of DPSFs was performed by using TGA (NETZSCH STA 449F5). The analysis was performed at different heating rates (10, 20, 30, and 40 °C/min). The analysis was performed using nitrogen as the inert medium at 60 mL/min.

### 2.4 Activation Energy (*E*_*a*_) Analysis

Model-free methods also known as the isoconversional method was used to estimate the *E*_a_ of DPSFs’ pyrolysis. Flynn–Wall–Ozawa (FWO), Kissinger–Akihara–Sunose (KAS), and Starink (STK) were the three model-free methods used in this study. The final eqs 1–3 after simplifications are^30^

1 $$KAS\;model:\;\ln\left(\frac{\beta }{T^{2}}\right)=\ln\left(\frac{A\times R}{E_{a}\times g\left(\alpha \right)}\right)-\frac{E_{a}}{RT}$$

2 $$OFW:\;OFW:\;\ln\left(\beta \right)=\ln\left(\frac{A\times E_{a}}{R\times g\left(\alpha \right)}\right)-5.331-1.052\left(\frac{E_{a}}{RT}\right)$$

3 $$STK:\;\ln\left(\frac{\beta }{T^{1.92}}\right)=C_{s}-1.008\left(\frac{E_{a}}{RT}\right)$$

In the KAS approach, a straight correlation will be attained by plotting ln­(β/*T* ^2^) against 1/*T*, where the slope corresponds to *E*_a_/*R*. In contrast, the FWO method depends on the linear plot of ln­(β) versus 1/*T*, yielding a slope equal to 1.052 *E*_a_/*R*. Likewise, the STK method establishes linearity by plotting ln­(β/*T* ^1.92^) against 1/*T*. α is the degree of conversion which is found using eq 4.^31^

4 $$\alpha =\frac{m_{0}-m_{i}}{m_{0}-m_{i}}$$

where *m*_0_ is the initial mass of the sample, *m*_i_ is the instantaneous mass of the sample, and *m*_f_ is the final mass of the sample. While using an isoconversional approach to calculate kinetics, α should be calculated on a constant *m*_f_ basis for all heating rates. The reason is that the isoconversional method compares temperatures at the similar conversion level in the calculation of kinetics. Otherwise, differences in the value of *m*_f_ can affect α and the estimated value of *E*_a_.

This study calculated the *E*_a_ based of *m*_f_ taken at 450 °C, where all four TGA curves converges to a common point to yield similar final mass. Within this temperature range lies the devolatilization/active pyrolysis region which is mainly responsible for the release of volatile components to produce bio-oil. After the devolatilization region (>450 °C), any discrepancies in the values of final residue for TGA curves at different heating rates will not affect the conversion values (α) and *E*_a_ estimation.

### 2.5 Pyrolysis of DPSFs and GC/MS Analysis of Bio-Oil

Pyrolysis of DPSFs was performed at optimized conditions using TGA and DTG analysis. Figure 2 shows a schematic assembly of the pyrolysis process utilized to produce bio-oil from DPSFs. DPSFs were pyrolyzed in a horizontal quartz tube flow reactor (SAFTherm, China). The reactor had a total length of 1400 mm with an internal diameter of 30 mm. The reactor was composed of two heating sections, each section extending 300 mm. The reactor was surrounded by a digitally controlled electric furnace to supply the required thermal energy. The furnace component offers accurate temperature regulation across the range of 5–1200 °C. The reactor inlet is connected to a *N*_2_ cylinder having a steady flow of 100 mL/min, while the outlet is attached to a PID controller and an ice trap fitted with an impinger bottle to capture volatile products. Further, the experimental conditions involved a nonisothermal heating with a ramp rate of 40 °C/min, beginning at 20 °C and progressing to 400 °C. Once the required set temperature was reached, the reactor was held isothermally at 400 °C for 20 min. Volatile compounds produced during pyrolysis, primarily within the 300–400 °C, were then condensed. The selection of this temperature range (20–400 °C) is based on the thermal degradation profile of DPSFs in TGA and DTG, which shows that the main active pyrolysis region for this lignocellulose biomass occurred in this temperature range. The condensable products were collected using an ice-cooled trap fitted with an impinger bottle containing dichloromethane to form a bio-oil.

**Figure 2:**
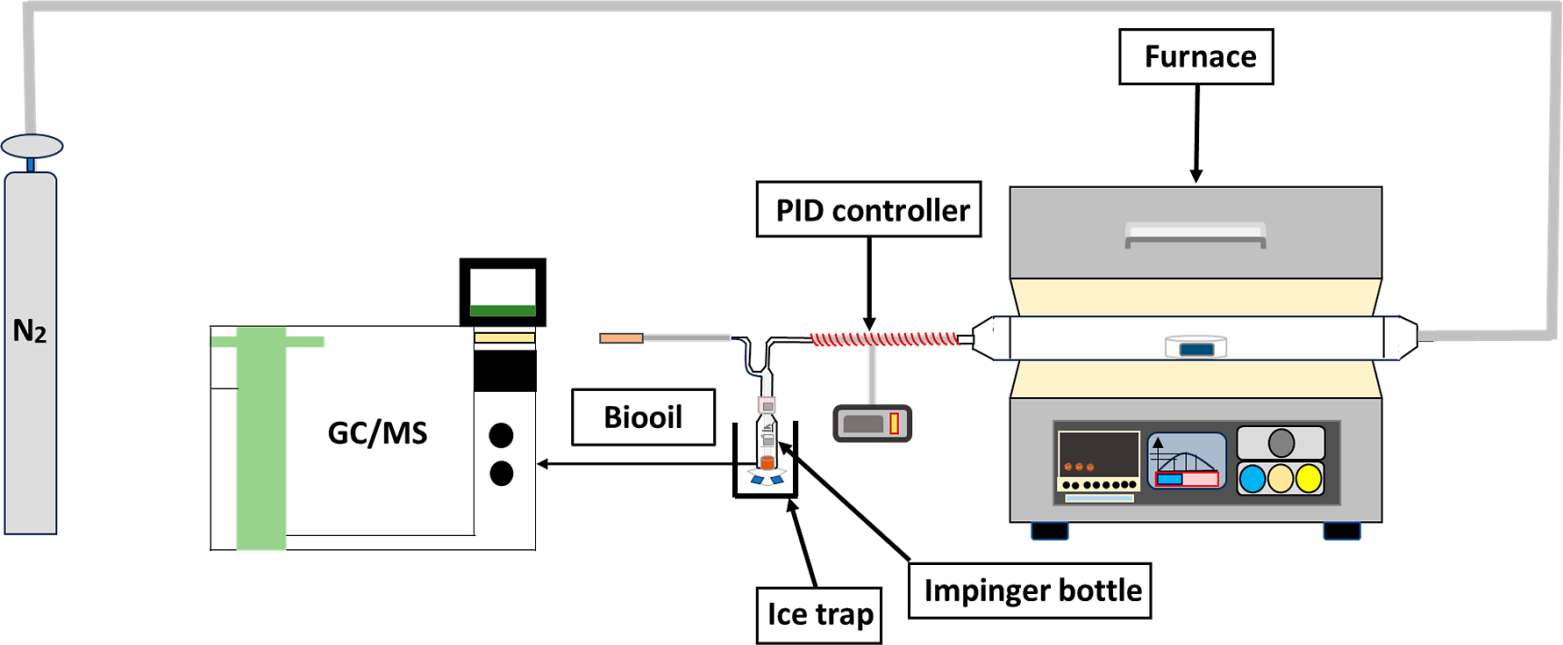
Schematic of DPSFs’ pyrolysis.

The bio-oil was analyzed using gas chromatography–mass spectroscopy (GC/MS) using a GC-MS 8890 from Agilent Technologies, USA. The bio-oil was mixed with acetone (1:50 v/v) and thoroughly homogenized using sonication (5 min). The bio-oil was also passed through a PTFE syringe filter to escape and solid contaminants. GC/MS analyses were performed using a split ratio of 10:1 with helium provided as the carrier gas at a constant flow rate of 1 mL/min. The HP-5MS-UI column was held at 250 °C, while the mass spectrometer interface was maintained at 300 °C. The oven temperature was set to rise from 50 °C at a rate of 15 °C/min. Compounds eluted from the GC column were detected by the mass spectrometer according to their molecular ion ratios (*m*/*z* 30–550). Later, the identification of the compounds was achieved by matching the spectra with those in the NIST library. The GC/MS composition was evaluated for instrumental repeatability through replicate injections of the same bio-oil sample to ensure the consistency of the compound percentages.

## 3 Results and Discussion

### 3.1 Proximate and Ultimate Analysis

Table 1 shows the proximate and ultimate analyses of DPSFs and other lignocellulosic biomass materials. The analysis represents significant insight into the energy potential of DPSFs. The higher amount of volatile matter (85.30%) and fixed carbon (9.20%) indicates an ample amount of condensable gas fractions for bio-oil production. Lower amount of ash content (2.90%) and lower moisture content (2.60%) also make DPSFs highly important as a pyrolysis feedstock. Ultimate analysis showed higher carbon (37.70%) and hydrogen (6.50%) content, which is suitable for high-energy fuels. The higher heating value (HHV) of DPSFs is 17.10 MJ/kg. The HHV of DPSFs is comparable to other lignocellulosic fuels such as raw sugar cane bagasse^32^ and wheat straw (17.62 MJ/kg).^33^ Compared to other regional date palm wastes, surface fibers had an HHV of 16.70 MJ/kg,^21^ date seeds had an HHV of 19.1 MJ/kg,^34^ and date leaf had an HHV of 16.88 MJ/kg.^35^ This implies that the energy input from surface fibers will be intermediate between that from seeds and that from other date biomass.

**Table 1:** Proximate and Ultimate Analysis of DPSFs

proximate analysis (dry mass fraction basis)	DPSFs this study	coconut shell^36^	sugar cane bagasse^37^	olive pits^38^
volatile matter (%)	85.30	77.19	83.66	82
fixed carbon (%)	9.20	22.10	13.15	16.28
ash content (%)	2.90	0.71	3.20	1.72
moisture (%)	2.60	-	-	-
ultimate analysis (dry mass fraction basis)				
C (%)	37.70	50.22	45.48	52.80
H (%)	6.50	5.70	5.96	6.69
N (%)	2.40	43.47	45.21	0.45
S (%)	-	-	-	
O (%)	53.40	-	-	38.25
HHV (MJ/kg)	17.10	20.50	18.73	21.59

### 3.2 Chemical Composition Analysis

Table 2 shows the chemical composition analysis of the DPSFs. The analysis shows that DPSFs are typical lignocellulose biomass, consisting of cellulose (48.20%), hemicellulose (24.30%), and lignin (27.50%). High cellulose content makes DPSFs an attractive feedstock for bio-oil production using pyrolysis technology. Compared to other DPSFs reported in the literature and similar lignocellulose materials, DPSFs in this have a higher cellulose content. High cellulose content suggests that DPSFs are attractive feedstock for bio-oil production using pyrolysis technology. Ansari et al.^39^ and Stefanidis et al.^40^ reported that the pyrolysis of cellulose is mainly responsible for producing bio-oil; however, the pyrolysis of lignin contributes to the formation of solid biochar.

**Table 2:** Chemical Composition of DPSFs

feedstock	cellulose	hemicellulose	lignin	ref
DPSFs	48.20 ± 5	24.30 ± 5	27.50 ± 5	this study
DPSFs	44.80	22.50	30.50	^21^
DPSFs	46	18	20	^41^
rice husk	35	30	18	^42^
date seeds	24.75	25.03	30.55	^43^
date leaves	41.90	29.50	21.40	^44^
ghaf waste	45.30	33.33	21.50	^45^

### 3.3 Thermodynamic Analysis

Figure 3 shows the thermal degradation profiles of DPSFs. The degradation mechanism is analyzed at four different heating rates (10, 20, 30, and 40 °C/min). DPSFs consisted of cellulose, hemicellulose, and lignin as the three main three biopolymers. Each of these constituents will bring a different contribution toward the pyrolysis behavior of DPSFs. Table 3 shows the thermal characteristics details of DPSFs at different heating rates. The pyrolysis degradation of DPSFs consisted of three distinct regions. The first region is termed the dehydration region, which involves the removal of moisture and light hydrocarbons. For DPSFs’ pyrolysis, this region comprises a temperature range of up to 268–290 °C. The second region is known as the devolatilization or the active pyrolysis region. The maximum mass-loss occurs in this region. For DPSFs’ pyrolysis, this region’s temperature range expands from 364 to 394 °C, resulting in a mass-loss of 56–61%. The degradation in this region of active pyrolysis is largely due to the degradation of cellulose and hemicellulose. Considering bio-oil production, the devolatilization region has the utmost importance because the volatile components are released in this region, which are later condensed to obtain bio-oil. The third region is known as the postpyrolysis or the carbonization region. In this region, degradation of remaining devolatilized biomass took place to form a carbon-rich material known as biochar.

**Figure 3:**
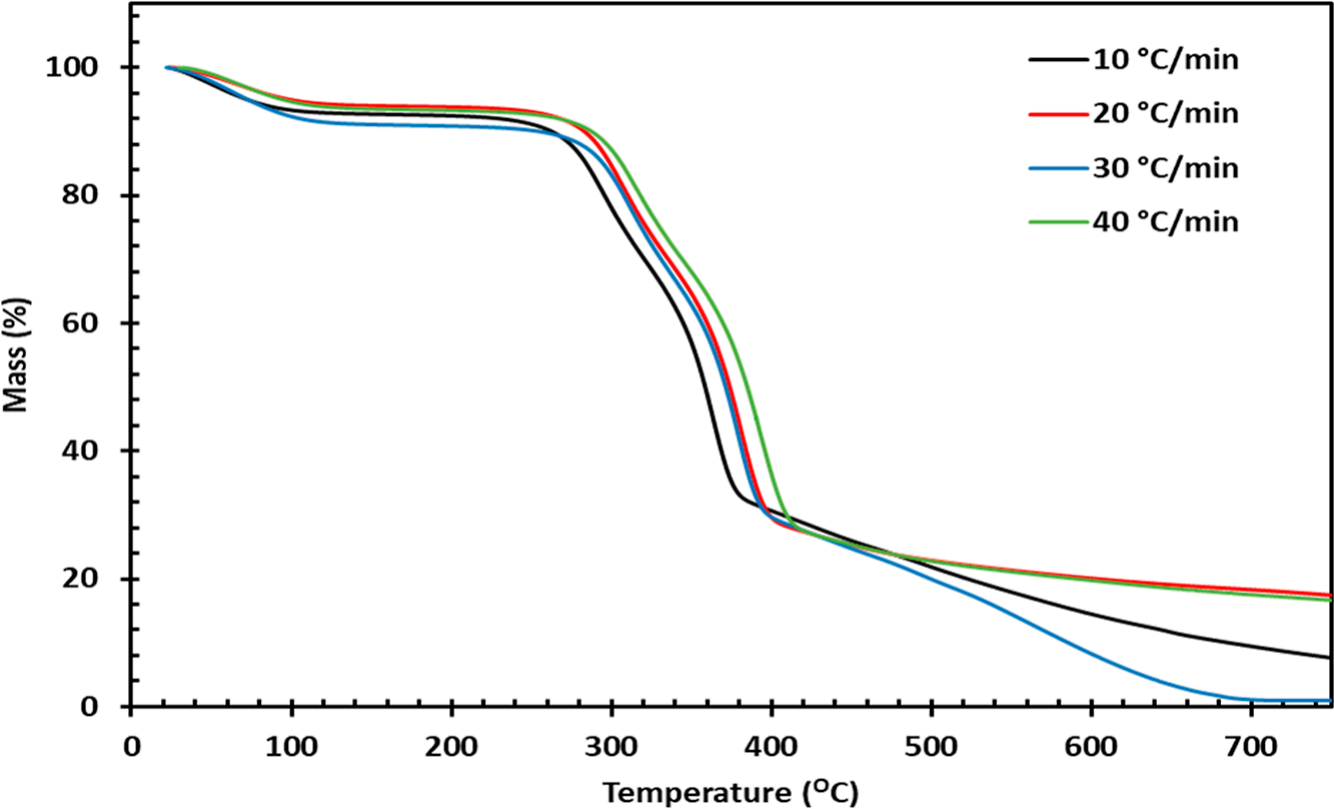
TGA of DPSFs at different heating rates.

**Table 3:** Thermal Characteristics of DPSFs’ Pyrolysis

heating rate (°C/min)	*T*_onset_(°C)	*T*_max_(°C)	mass_loss_	DTG peak (°C)
10	268	380	56	364
20	279	398	61	384
30	285	393	56	379
40	290	414	61	394

Figure 4 shows the differential thermogravimetric analysis (DTG) of DPSFs’ pyrolysis at different heating rates (10, 20, 30, and 40 °C/min). DTG peaks refer to the temperature at which the maximum mass-loss occurs during pyrolysis. For DPSFs’ pyrolysis, the DTG peaks were in a temperature range of 364–394 °C. The TGA and DTG analyses of DPSFs’ pyrolysis at different heating rates show that a temperature range of 270–400 °C, with a heating rate of 20 °C/min, with a maximum residence temperature of 380 °C, is the most feasible pyrolysis condition to release about 60% of the lignocellulose biomass into volatiles. Previous investigations on lignocellulose biomass pyrolysis have shown that increasing the pyrolysis temperature increases the bio-oil yield up to an intermediate liquid-max window, increases the noncondensable gas yield, and decreases the biochar yield. Also, an increase in the heating rate decreases biochar yield and increases volatile content for bio-oil production.^46–48^

**Figure 4:**
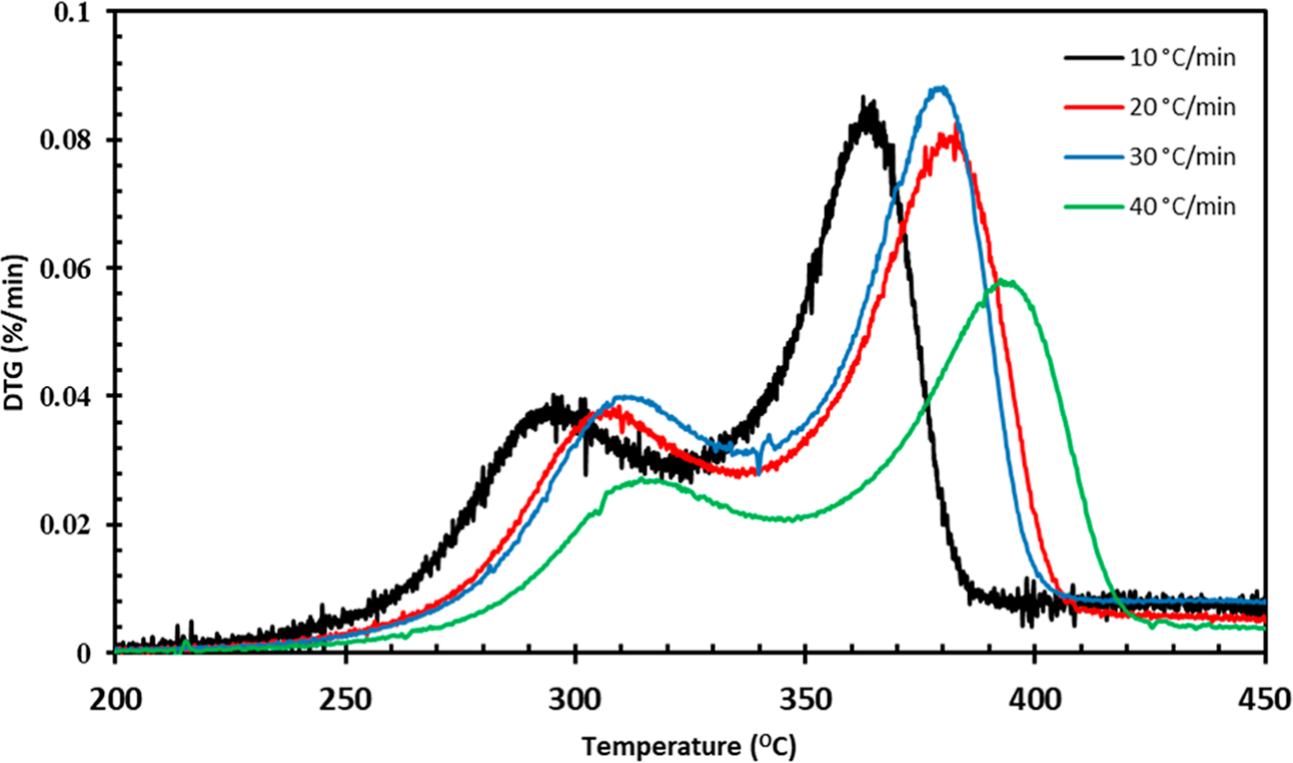
DTG analysis of DPSFs at different heating rates.

### 3.4 Activation Energy (*E*_*a*_) Analysis

*E*_a_ analysis helps determine the minimum energy requirement to initiate the degradation of DPSFs’ components (cellulose, lignin, and hemicellulose). *E*_a_ helps determine which components are more reactive and at what temperatures the major degradation will occur.^49^ Particularly, *E*_a_ analysis helps determine the most energy efficient temperature window for pyrolysis reactions. This information on *E*_a_ further needed for the efficient design of pyrolysis reactors.^50^

Model-free kinetic approach was used to determine the *E*_a_ of DPSFs’ pyrolysis using Ozawa–Flynn–Wall (OFW), Kissinger–Akahira–Sunose (KAS), and Starink (STK) models. Model-free kinetics determines *E*_a_ as a function of conversion (α).^51^ If *E*_a_ varies with conversion, it shows that it is a multistep pyrolysis reaction. Conversion values below 0.2 and above 0.8 were excluded due to quite lowered linearity (lower *R* ^2^) at the extremes. Moreover, the interest of this study was bio-oil production; therefore, the conversion range between 0.2 and 0.8 is the active pyrolysis region mainly responsible for producing the volatiles. The variation of *E*_a_ with conversion shows that pyrolysis of DPSFs is a multistep process mainly due to the overlapping degradation of cellulose, hemicellulose, and lignin. At a low conversion range (α = 0.2–0.35), the increasing trend of *E*_a_ values is due to the onset of devolatilization. In this region, hemicellulose starts to degrade and easier to cleave side chains become to break. This region is in a temperature range of 220–315 °C. At the intermediate conversion values (α = 0.35–0.5), the *E*_a_ values are almost steady with very negligible increase. In this region, the decomposition of cellulose takes place. This region dominantly includes release of volatiles in a temperature range of 315–400 °C. In a higher conversion range (α = 0.5–0.8), the increase in *E*_a_ is due to accelerated fragmentation of lignin and char-forming reactions. Lignin decomposes over a broad spectrum and, therefore, includes complex bond breaking pathways. Figure 5a–c shows the kinetic plots for the KAS, OFW, and STK models using experimental TGA data at specific degrees of conversion (αi). By repeating this model fitting process at different conversions, we obtained a profile of *E*_a_ as a function of conversion was obtained. Figure 5d shows that *E*_a_ is the same function of conversion for all three models. Table 4 shows the values of *E*_a_ and the regression coefficient (*R* ^2^) for KAS, OFW, and STK models. The average values of *E*_a_ using KAS, OFW, and STK were 152.40, 154.52, and 152.37 kJ/mol, respectively. *E*_a_ values from all three models were close and therefore signify the accuracy of the estimated values. Cai and Bi^52^ estimated the effective *E*_a_ energy for wheat straw to be 130–175 kJ/mol, using the OFW model. Mishra and Mohanty^53^ estimated the effective *E*_a_ energy for waste sawdust to be 171.66 kJ/mol, using KAS model. Nawaz and Kumar^54^ estimated the effective *E*_a_ energy for mustard straw to be 202.19 kJ/mol, using the OFW model. Kumar et al.^55^ estimated the effective *E*_a_ energy for sugar cane bagasse to be 226.97 kJ/mol, using the OFW model. The estimated effective *E*_a_ energy for DPSFs in this study is approximately 152 kJ/mol, which shows the structure integrity of the fiber’s matrix and higher crystallinity of cellulose fibers.

**Figure 5:**
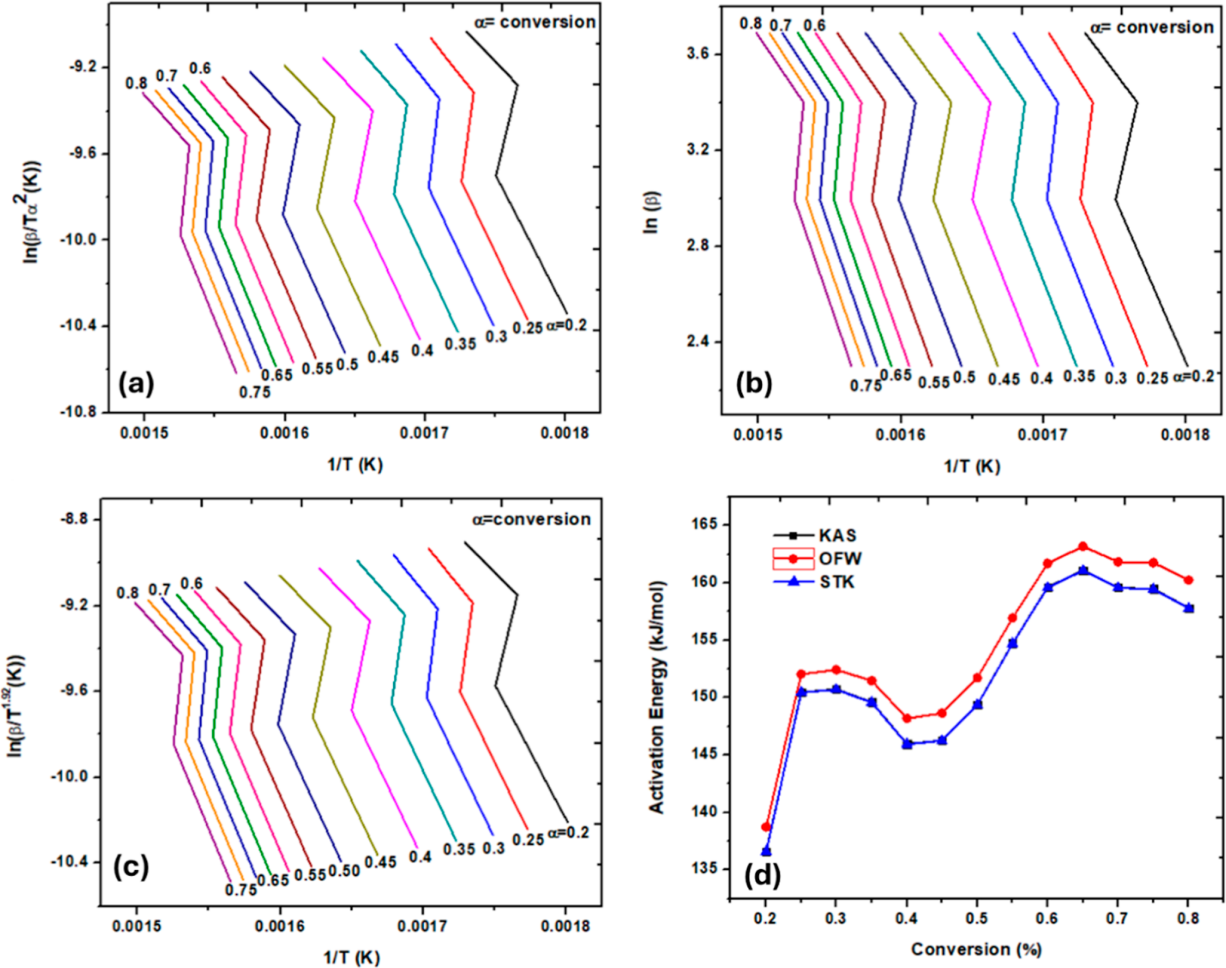
Kinetic plots for DPSFs’ pyrolysis using the (a) KAS model, (b) OFW model, and (c) STK model and (d) *E*_a_ as a function of conversion (%).

**Table 4:** *E*_a_ Values Calculated Using KAS, OFW, and STK Models

Α	KAS		OFW		STK	
	*E*_a_ (kJ/mol)	*R* ^2^	*E*_a_ (kJ/mol)	*R* ^2^	*E*_a_ (kJ/mol)	*R* ^2^
0.2	136.57	0.75	138.71	0.77	136.56	0.72
0.25	150.45	0.85	152.04	0.84	150.44	0.82
0.3	150.73	0.83	152.42	0.85	150.70	0.84
0.35	149.58	0.81	151.47	0.83	149.56	0.82
0.4	145.94	0.77	148.17	0.79	145.93	0.77
0.45	146.25	0.77	148.62	0.79	146.23	0.77
0.5	149.35	0.77	151.72	0.8	149.34	0.78
0.55	154.71	0.8	156.93	0.82	154.69	0.8
0.6	159.61	0.82	161.69	0.84	159.59	0.82
0.65	161.09	0.83	163.17	0.85	161.07	0.84
0.7	159.59	0.84	161.82	0.85	159.58	0.84
0.75	159.46	0.84	161.75	0.85	159.44	0.84
0.8	157.79	0.83	160.24	0.85	157.78	0.83
avg	152.40		154.52		152.37	

### 3.5 GC/MS Analysis of Bio-Oil

Figure 6 shows the distribution of compound families found in DPSF-derived bio-oil by GC/MS. The bio-oil was mainly composed of aliphatics (42.28%), aromatics (38.68%), and furans/other oxygenates (13.47%), while the remaining 5.57% links to trace or unassigned peaks (e.g., below the identification/quality threshold). All percentages are reported as normalized GC/MS peak-area percentages (semiquantitative). Table 5 lists the identified compounds in detail. 4-hydroxy-4-methyl-2-pentanone, which is a hydroxy-ketone, appears as the major component in aliphatic compounds, with a dominant share of 34.28%. Other minor aliphatics include alkenes (2-heptene, 1-octene, 1-undecene), alcohols (1-dodecanol), and ketones (3-hexene-2-one). The presence of hydroxyketones in aliphatic compounds suggests strong carbohydrate (cellulose and hemicellulose) decomposition through concurrent transglycosylation, dehydration, and open ring/fragmentation routes. This phenomenon generates light oxygenates and furanic precursors.^56^ The aromatic compounds were mainly dominated by toluene (10.94%), benzene (10.54%), *p*-xylene (3.87%), phenol (2.34%), and indene (2.03%). Benzene, toluene, and xylene, referred to as BTX fractions, are industrially important as high-octane fuel additives and petrochemical intermediates. Aromatic compounds result from the pyrolysis of lignin in the lignocellulosic biomass through cleavage of ether linkages and side-chain reactions (e.g., demethoxylation/demethylation/decarbonylation).^57^ Main compounds in furan derivatives were furfural (6.73%), 2,5-dimethylfuran (2.12%), and 2-furanmethanol (1.80%). Furans appear as a result of the pyrolysis of hemicellulose in the lignocellulosic biomass. Furan compounds (furfural, furfuryl alcohol, dimethylfuran) can be rationalized by dehydration chemistry of hemicellulose-derived C_5_ sugars followed by secondary transformations (hydrogen transfer/deoxygenation) in the vapor phase.^58^ Nearly 40% aromatics is remarkably high for bio-oil, making DPSFs’ pyrolysis mainly favorable for BTX and phenolic recovery. The detection of BTX (benzene, toluene, xylene) together with benzofuran and light PAHs (indene, naphthalene) suggests that a portion of primary oxygenates/phenolics undergo secondary cracking, cyclization, and aromatization/condensation during hot-vapor transport and quenching. This is a common phenomenon that is well-known to influence to the final GC/MS-observed slate.^59^ Since benzene is classified as a carcinogen, BTX can pose health and environmental risks during fuel use. However, in fuel applications, these concerns are mitigated by regulatory limits and emission control. Furthermore, catalytic pyrolysis of such feedstocks can be performed to improve the fuel stability and efficiency.^60^ In addition, the oxygenated compounds are known to be corrosive due to their lower pH, which can cause problems during storage and handling. The reactive oxygen species also limit long-term storage, as they cause stability changes and an increase in viscosity. Mitigation can be done in two ways: either by upgrading to reduce acids and reactive carbonyls, or by using corrosion-resistant containers and storage.^61^

**Figure 6:**
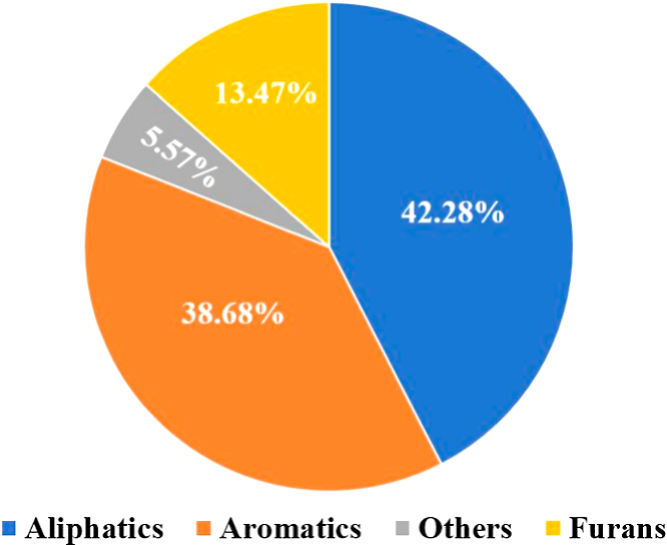
Percentage of fractions from DPSFs’ bio-oil.

**Table 5:** Components Identified in the GC/MS Analysis of Bio-Oil Produced from DPSFs

components	formula	*M*_W_ (g/mol)	peak %
aliphatics			
2- heptene	C_7_H_14_	98.19	0.82
1-octene	C_8_H_16_	112.21	1.08
3-hexene-2-one	C_6_H_10_O	98.14	3.68
4-hydroxy-2-pentanone	C_5_H_10_O_2_	102.13	1.08
4-hydroxy-4-methyl-2-pentanone	C_6_H_12_O_2_	116.16	34.28
1-methyl-2-pentyl-cyclopropanone	C_9_H_16_O	140.23	0.64
1-undecene	C_11_H_22_	154.29	1.61
1-dodecanol	C_12_H_26_O	186.33	0.49
aromatics			
benzene	C_6_H_6_	78.11	10.54
toluene	C_7_H_8_	92.14	10.94
ethylbenzene	C_8_H_10_	106.16	1.56
p-xylene (1,4-dimethylbenzene)	C_8_H_10_	106.16	3.87
styrene (vinylbenzene)	C_8_H_8_	104.15	1.9
phenol	C_6_H_6_O	94.11	2.34
1-ethenyl-2-methylbenzene	C_9_H_10_	118.18	1.2
indene	C_9_H_8_	116.16	2.03
p-cresol (4-methylphenol)	C_7_H_8_O	108.14	2.25
2-methyl-indene	C_10_H_10_	130.19	0.57
naphthalene	C_10_H_8_	128.17	1.48
furans			
2,5-dimethyl-furan	C_6_H_8_O	96.13	2.12
furfural (2-furaldehyde)	C_5_H_4_O_2_	96.08	6.73
2-furanmethanol (furfuryl alcohol)	C_5_H_6_O_2_	98.10	1.8
benzofuran	C_8_H_6_O	118.13	0.97
5-methyl-2-furancarboxaldehyde	C_6_H_6_O_2_	110.11	1.85

Hai et al.^62^ performed fast pyrolysis of date palm seeds and date palm syrup from industrial waste. The bio-oil produced was examined by using GC/MS analysis. The bio-oil mainly consisted of furanics and sugar-derived oxygenate. The most important components in the bio-i-oil from date seeds were furfuryl alcohol (∼21%), *d*-allose (∼19.6%), catechol (∼7.9%), 1-cyclopropylpentane (∼6.8%), furfural (∼5.1%), and 5-hydroxymethylfurfural (∼4.5%). The bio-oil from date syrup waste consisted of mainly 2-fluoroformyl-3,3,4,4-tetrafluoro-1,2-oxazetidine (∼31%). Date palm seed bio-oil does not show an aromatic/BTX dominant composition. This suggests that the pyrolysis of DPSFs supports the greater production of lighter aromatics through lignin fragmentation and secondary vapor-phase aromatization.

## 4 Discussion and Future Perspectives

In this study, low-value biomass waste was converted into energy applications using pyrolysis technology. For the disposal of lignocellulose biomass wastes, an expensive waste management system is required. Otherwise, they are burned in agricultural fields, which causes the release of particulate matter and gaseous pollutants that affect the environment.^63^ For example, postharvest burning in the agricultural fields of the Punjab regions of Pakistan and India generates smog during the winter season, which causes respiratory problems.^64^ Lignocellulose biomass utilization itself is carbon neutral, as it only releases the carbon it has utilized from the atmosphere throughout the photosynthesis process during its growth.^65^ Controlled thermochemical conversion, such as pyrolysis, even makes its utilization beneficial with respect to carbon footprint.^66^ Different authors have also performed the techno-economic analysis of lignocellulosic biomass wastes. Parthasarathy et al.^67^ performed the techno-economic analysis of date stones’ pyrolysis using conventional and microwave-assisted pyrolysis. The findings suggest that conventional pyrolysis is superior in terms of profitability, financial stability, and overall success. Shabangu et al.^68^ performed the pyrolysis using simulated software. They concluded that together with biofuel, biochar as a marketable product is important for the economic feasibility of the process. Amjed et al.^69^ performed the techno-economic analysis of solar driven bio-oil and biochar production for improving the carbon efficiency. They concluded that the bio-oil cost is highest if the pyrolysis plant is operated with no biochar yield. Fadhilah et al.^70^ performed the techno-economic analysis of sawdust and rice husk copyrolysis for bio-oil production. This study showed that the operating labor costs, nitrogen consumption, and feedstock costs significantly influence the unit production costs of bio-oil.

There are certain limitations of this work including yield calculations of bio-oil, biochar, and noncondensable gases. The mass and energy balances of the pyrolysis process advocate its efficiency. It is therefore suggested that future research studies should be focused on resolving these shortcomings and performing the techno-economic analysis. In addition to this, catalytic pyrolysis of DPSFs can be performed to improve the yield and composition of bio-oil.

## 5 Conclusion

Pyrolysis of DPSFs was performed using a horizontal quartz tube flow reactor nonisothermally at a heating rate of 40 °C/min and a temperature range of 20–400 °C. TGA and DTG were performed at 10–40 °C/min to identify the optimized pyrolysis conditions. *E*_a_ as a function of conversion was estimated to be 152 kJ/mol using three isoconversional methods including Ozawa–Flynn–Wall (OFW), Kissinger–Akahira–Sunose (KAS), and Starink (STK) models. GC/MS analyses showed that the bio-oil is enriched with aliphatics (42.28%), aromatics (38.68%), and furans (13.47%). The information on the DPSF pyrolyzed bio-oil suggests that the aromatic rich nature will lead to targeted recovery of BTX/phenolic compounds, as well as for bioenergy applications.
